# An Albumin, Neutrophil, and Lymphocyte-Related Risk Estimation Tool in Hospitalised Patients

**DOI:** 10.7759/cureus.64197

**Published:** 2024-07-09

**Authors:** Ethar N Ibrahim, Hisham A Alrashdan, Osama Alshiyyab, Zuhier A Ikhwayleh, Samer Alboun, Abedal-Rahman I Al-Theiabat, Ali F Al-Shatnawi, Mohammad T Aldeeb, Yarub M Almiqdad, Mino Cycline

**Affiliations:** 1 Anaesthesia Department, Royal Medical Services, Irbid, JOR; 2 Otolaryngology - Head and Neck Surgery Department, Jordanian Royal Medical Services, Amman, JOR; 3 Emergency Department, Jordanian Royal Medical Services, Amman, JOR; 4 Rehabilitation and Rheumatology Department, Jordanian Royal Medical Services, Amman, JOR; 5 Internal Medicine Department, Jordanian Royal Medical Services, Amman, JOR; 6 Research and Development Department, Jordanian Royal Medical Services, Amman, JOR

**Keywords:** adverse clinical outcomes, hospitalized patient, risk assessment tools, neutrophil albumin ratio, neutrophils to lymphocytes ratio

## Abstract

Aim: The neutrophil-to-lymphocyte ratio (NLR) is commonly used as a prognostic indicator for microbiological and inflammatory conditions in clinical settings. However, the quotient to albumin levels, which is another nutritional and clinical predictor, may also have an interesting diagnostic and prognostic value. This study aimed to primarily investigate the predictive performances of the neutrophils to albumin and lymphocytes ratio (NALR) compared to the NLR in predicting poor outcomes during hospital admission, particularly the decomposition of respiratory, renal, liver, and circulatory systems, resulting in longer hospital stays or mortality.

Methods: An observational study was performed on a cohort of 270 hospitalised patients admitted to Rashid bin Al-Hussein Military Hospital during the period from October 2023 to early November 2023. The study specifically targeted adult patients (age >17 years) who had a minimum of 80% availability of their initial and follow-up data during admission. We dichotomised all eligible test patients into two groups: Group I, which represented better outcomes of interest, and Group II, which represented poorer outcomes of interest. Statistically, we conducted binary logistic, receiver operating, and sensitivity analyses to explore the predictive performances and indices for NALR and NLR. We also conducted chi-square and independent T analyses to uncover the distribution rates of the independent variables across Groups I and II. We considered a p-value of less than 0.05 as the level of significance.

Results: Out of a total sample size of 270, 82 patients (30.37%) were allocated to Group I, and 188 patients (69.63%) were allocated to Group II. Males outnumbered females in this study by 184 (68.1%) to 86 (31.9%). Patients in the study had an average age of 58.08±10.02 years. The average hospitalisation took 13.71±6.38 days, significantly longer in Group II compared to Group I (15.43±6.76 days vs. 9.77±2.69 days, p-value<0.05). We found that the area under the receiver operating characteristic (ROC) curves was estimated at [0.808±0.031 (0.748-0.868), p-value=0.000] and [0.667±0.034 (0.601-0.733), p-value=0.000] for NALR and NLR, respectively. The optimal operating thresholds for NALR and NLR were 1.5 and 5.37, with sensitivities and specificities of 86.7% versus 73.4% and 70.73% versus 70.73%, respectively.

Conclusion: The proposed NALR showed superior predictive performance, sensitivity, and correlation compared to the parent NLR. Both tools can be used in clinical practice to prioritise clinical and pharmacotherapeutics for hospitalised patients based on unfavourable outcomes.

## Introduction

Hospitalised patients undergo routine hematologic and biochemical blood tests for albumin, neutrophils, and lymphocytes for diagnostic and prognostic purposes. For example, the Model for End-Stage Liver Disease (MELD) score predicts cirrhosis patients' three-month survival rates using albumin and neutrophils [1]. The blood urea nitrogen-to-albumin ratio (BAR) is used to assess renal function in chronic kidney disease patients, and the albumin-bilirubin (ALBI) score, a recently developed highly responsive model for predicting end-stage liver disease, is another practical instance [2].

However, the levels of neutrophils and lymphocytes are not typically overlooked, except when evaluating immune function or as markers for particular hematologic or autoimmune disorders. Neutrophils constitute the majority of white blood cells, comprising over 50% of the overall count. Macrophages play a pivotal role in the innate immune system as they serve as the initial barrier against pathogens [3]. Neutrophils use surface receptors to recognise and adhere to targets and contain bactericidal enzymes in their diapedesis granules and nucleus to kill bacteria. Neutrophil lysosomes kill pathogens and reduce tissue damage from neutrophil inflammation. However, neutrophils can worsen tissue damage, which is a clinical sign of elevated neutrophil levels even without an infection [4].

The neutrophil-to-lymphocyte ratio (NLR) predicts the stone score used to determine kidney stone surgery and bacterial-associated systemic inflammatory response syndromes (SIRS). These cases have an NLR 6-7 times higher than non-infection-related SIRS. Using this ratio in hospitalised patients is risky because corticosteroidal agents for other indications tend to exaggerate it [5]. The NLR was also tested in cancer patient studies. Prediction models consistently showed that the NLR is associated with poor cancer outcomes. However, these studies mostly looked back at past data, examined only one organ, and included a wide range of patients [5].

The adaptive immune system relies on lymphocytes, which remember foreign antigens. When they encounter the same antigen again, they can synchronise a faster and stronger immune response [6]. B-cells have the ability to directly fight pathogens using antibodies produced by immunoglobulin genes, while T-cells can either kill infected cells that present antigenic peptide fragments in major histocompatibility complex (MHC) molecules or stimulate B-cells to produce more antibodies. Lymphocytes' ability to control internal and external tissue damage, especially neutrophil-induced tissue damage, distinguishes them in risk estimation models [7]. The differential count of monocytes, eosinophils, and basophils is less than 10%. However, monocytes and neutrophils play different adaptive immune roles. Hospitalisation can be predicted using NLR, age, and hematocrit. It is important to note that each hospital may use different variables to assess disease severity [8].

Albumin, which has been used for nutritional assessment and clinical risk [9], when added to the NLR, a new composited prognosticator (neutrophils to albumin and lymphocytes ratio, NALR), may be a reliable and autonomous indicator for treatment efficacy and patient outcomes, or even better than its parent ratio (NLR) at predicting hospitalisation and clinical outcomes of admitted patients [10]. Hospitalisation was based on illness severity, and the NALR and NLR were used to assess prognosis and hospital stay. The NALR validation study found that this multifactor index outperforms the pneumonia severity index and others. Multiple studies have examined the NLR's prognostic value for hospitalisation outcomes. The NALR was compared to the parent NLR in this study [11].

The NLR is commonly used as a prognostic indicator for microbiological and inflammatory conditions in clinical settings. However, the quotient to albumin levels, which is another nutritional and clinical predictor, may also have an interesting diagnostic and prognostic value [12].

This study aimed to primarily investigate the predictive performances of the NALR compared to the NLR in predicting poor outcomes during hospital admission, particularly the decomposition of respiratory, renal, liver, and circulatory systems, resulting in longer hospital stays or mortality.

## Materials and methods

Our retrospective-observational study included 270 medical and surgical inpatients admitted between 1 October 2023 and 5 November 2023 in Rashid bin Al-Hussein Military Hospital, Irbid governorate, Jordan. This study was approved by the Jordanian Institutional Review Board (IRB) at the Royal Medical Services (RMS) under registration number 41_4.2024 on 14/5/2024. Only adult patients aged ≥18 years and who had been admitted for at least 48 hours but no more than 96 hours were included in this study, as long as their missing data did not exceed 20% for each.

Sex, age, hospital arrival and discharge date, pre-existing comorbidity burden, death date, and biochemical results were retrieved from our institutional electronic data information. This study focused on biochemical tests such as albumin level (g/dl), lymphocyte count (cells/µl), and neutrophil count (cells/µl). An adverse outcome of interest (OI) included the development of acute respiratory distress syndrome, acute kidney injury, liver dysfunction worsening, hemodynamic instability worsening, transfer to the critical care unit, longer hospital stays than expected, or death. Most of the patients’ collected data were retrieved from our institutional electronic recording system (Hakeem). A positive condition in this study was the presence of poorer OI (signed as 1) while the negative condition in this study was the presence of better OI (signed as 0).

The surgical procedures performed on our tested patients who were admitted for surgery were primarily evaluated and categorised into the following types: colorectal surgery, appendectomy, Whipple procedure, gastric sleeve surgery, and osteotomy closure. All eligible patients who were studied were admitted to our institutional wards, either in the surgical or medical departments.

We dichotomised all eligible tested patients, in this study, into two groups: Group I, which represented better OIs, and Group II, which represented poorer OIs. The tested independent variables were compared between two comparative groups: the better OI, or negative state, and the poorer OI, or positive state. We used the chi-square test to compare the distribution rates (numbers with percentages) between groups and to abstract the related Pearson correlations, odd ratios, and p-values. In contrast, the parametric independent variables were compared across Groups I and II by conducting independent t-tests to express the results as means with its standard deviations and mean differences. One-sample t-test was used to present the whole cohort as mean±SD.

As mentioned, this study examined the effectiveness and predictive power of the proposed NALR and its parent NAL prognosticators against OI rates. To achieve these intended purposes, we used binary logistic regression to compare the NALR and NLR with the likelihood of poorer outcomes and the variability ranges for the quality of prediction. Also, we conducted a receiver operating characteristic (ROC) test to evaluate the area under the curve (AUC) values and subsequently, we pursued sensitivity analyses to explore the optimal thresholds in adjunct to other sensitivity indices.

Microsoft Excel was used to collect and organise the patient data. IBM SPSS Statistics 25 (IBM Corp, Armonk, NY) was used for statistical analysis and study summary. This study used 0.05 significance.

## Results

Out of a total sample size of 270, 82 patients (30.37%) were allocated to Group I and 188 patients (69.63%) were allocated to Group II. Males outnumbered females in this study by 184 (68.1%) to 86 (31.9%). The distribution of tested genders across Group I to Group II was not statistically significant [0.596 (95% CI: 0.332-1.072), p-value>0.05]. However, the number of males tested in this study was approximately more than twice that of females tested [184 (68.1%) vs 86 (31.9%), respectively].

The average age of the patients included in the study was 58.08±10.02 years, with a mean difference of -4.788±1.295 (95% CI: -7.338 to -2.238) years (Group II: 59.53±10.86 years versus Group I: 54.74±6.68 years).

This study aimed to include both medically and surgically treated patients. However, there were no significant differences in the distribution rates between the two groups [0.832 (95% CI: 0.470-1.472), p-value>0.005]. The number of surgically tested patients was almost twice as high as the number of medically tested patients in both Group I and Group II [59 (72.0%) vs 23 (28.0%) and 128 (68.1%) vs 60 (31.9%), respectively].

Statistically significant differences were found in the prevalence of comorbidities between the poorer OI group (Group II) and the better OI group (Group I). Specifically, 41 (21.8%) and 42 (22.3%) individuals in Group II had a comorbidity burden of 3 and 4, respectively, compared to 42 (51.2%) and 40 (48.8%) individuals in Group I with a comorbidity burden of 1 and 2, respectively. In this study, we examined a statistically significant moderate positive Pearson correlation (+0.486±0.036) between an increased burden of comorbidity and an increased likelihood of experiencing the OIs.

The average length of hospitalisation was found to be 13.71±6.38 days, which was significantly longer in Group II compared to Group I (15.43±6.76 days versus 9.77±2.69 days, respectively, p-value<0.05) with a mean difference of -5.657±0.772 (95% CI: -7.178 to -4.137). Upon analysis, we found a statistically significant positive correlation (Pearson correlation coefficient: +0.417±0.030, p-value<0.05) between staying in the hospital for more than 14 days and experiencing poorer OIs. However, the majority of the patients included in the study had a duration of stay that was less than 14 days, as opposed to more than 14 days [193 (71.5%) vs 77 (28.5%), respectively].

From a biochemical perspective, the poorer OI group (Group II) had lower albumin levels (3.552±0.659 g/dl) and total lymphocyte count (TLC; 1531.4±1434.0 cells/µl) compared to the better OI group (Group I) with albumin levels of 3.77±0.559 g/dl and TLC of 1571.0±723.3 cells/µl. The p-values for the differences were 0.016 and 0.000, respectively. The mean differences were 0.216±0.084 (95% CI: 0.051 to 0.380) for albumin levels and 39.596±167.034 (95% CI: -289.27 to 368.46) for TLC. 

In contrast, Group II exhibited significantly higher levels of absolute neutrophils count, NLR, and NALR compared to Group I. The values for Group II were 9624.2±3762.8 cells/µl, 9.6599±4.860, and 2.607±0.997, respectively, while for Group I, they were 8365.1±2080.2 cells/µl, 6.649±3.06121, and 1.732±0.666, respectively. The mean differences between Group I and Group II were -1259.1±442.6 (95% CI: -2130.6 to -387.6), -3.01±0.582 (95% CI: -4.155 to -1.87), and -0.876±0.120 (95% CI: -1.11 to -0.638), respectively. All of the aforementioned comparative analyses’ results are thoroughly described in Tables 1, 2.

**Table 1:** Comparatively studied independent parametric variables across the dichotomised adverse outcomes of interest-based cohorts I-II. LOS: length of stay; OI: outcomes of interest; TLC: total lymphocytes count; ANC: absolute lymphocytes count; NLR: neutrophils-to-lymphocytes ratio; NALR: neutrophils to albumin and lymphocytes ratio; OR: odds ratio; CI: confidence interval; LL: lower limit of the confidence interval; UL: upper limit of the confidence interval.

	Group I Better OI, Negative State (82, %)	Group II Poorer OI, Positive State (188, %)	Total (270, 100%)	Mean Differences±SEMs (95% CI; LL-UL)	p-Value
Age (years)	54.74±6.68	59.53±10.86	58.08±10.02	-4.788±1.295 (95% CI: -7.338 to -2.238)	0.000
LOS	9.77±2.69	15.43±6.76	13.71±6.38	-5.657±0.772 (95% CI: -7.178 to -4.137)	0.000
Albumin (g/dl)	3.77±0.559	3.552±0.659	3.62±0.638	0.216±0.084 (95% CI: 0.051 to 0.380)	0.016
TLC (cells/µl)	1571.0±723.3	1531.4±1434.0	1543.43±1259.9	39.596±167.034 (95% CI: -289.27 to 368.46)	0.000
ANC (cells/µl)	8365.1±2080.2	9624.2±3762.8	9241.79±3388.5	-1259.1±442.6 (95% CI: -2130.6 to -387.6)	0.000
NLR	6.649±3.06121	9.6599±4.860	8.75±4.601	-3.01±0.582 (95% CI: -4.155 to -1.87)	0.000
NALR	1.732±0.666	2.607±0.997	2.34±0.994	-0.876±0.120 (95% CI: -1.11 to -0.638)	0.000

**Table 2:** The study conducted a comparative analysis of independent categorical variables across the dichotomized adverse outcomes of interest-based cohorts (I-II). F: female gender; M: male gender; LOS: length of stay; OI: outcomes of interest; OR: odds ratio; R: Pearson correlation. ^#^Number of comorbidities or burden. *p<0.05.

	Group I Better OI, Negative State (82, 30.37%)	Group II Poorer OI, Positive State (188, 69.63%)	Total (270, 100%)	OR	R	p-Value
Gender						
F	20 (24.4%)	66 (35.1%)	86 (31.9%)	0.596 (95%; 0.332-1.072)	-0.106±0.058	0.082
M	62 (75.6%)	122 (64.9%)	184 (68.1%)			
Department						
Medical	23 (28.0%)	60 (31.9%)	83 (30.7%)	0.832 (95% CI: 0.470-1.472)	-0.039±0.060	0.527
Surgical	59 (72.0%)	128 (68.1%)	187 (69.3%)			
Comorbidity^#^						
1	42 (51.2%)	25 (13.3%)	67 (24.8%)	NA	+0.486±0.036*	0.000*
2	40 (48.8%)	80 (42.6%)	120 (44.4%)			
3	0 (0.0%)	41 (21.8%)	41 (15.2%)			
≥4	0 (0.0%)	42 (22.3%)	42 (15.6%)			
LOS days						
<14	82 (100.0%)	111 (59.0%)	193 (71.5%)	0.575 (95% CI: 0.509-0.649)	+0.417±0.030*	0.000*
≥14	0 (0.0%)	77 (41.0%)	77 (28.5%)			

**Figure 1:**
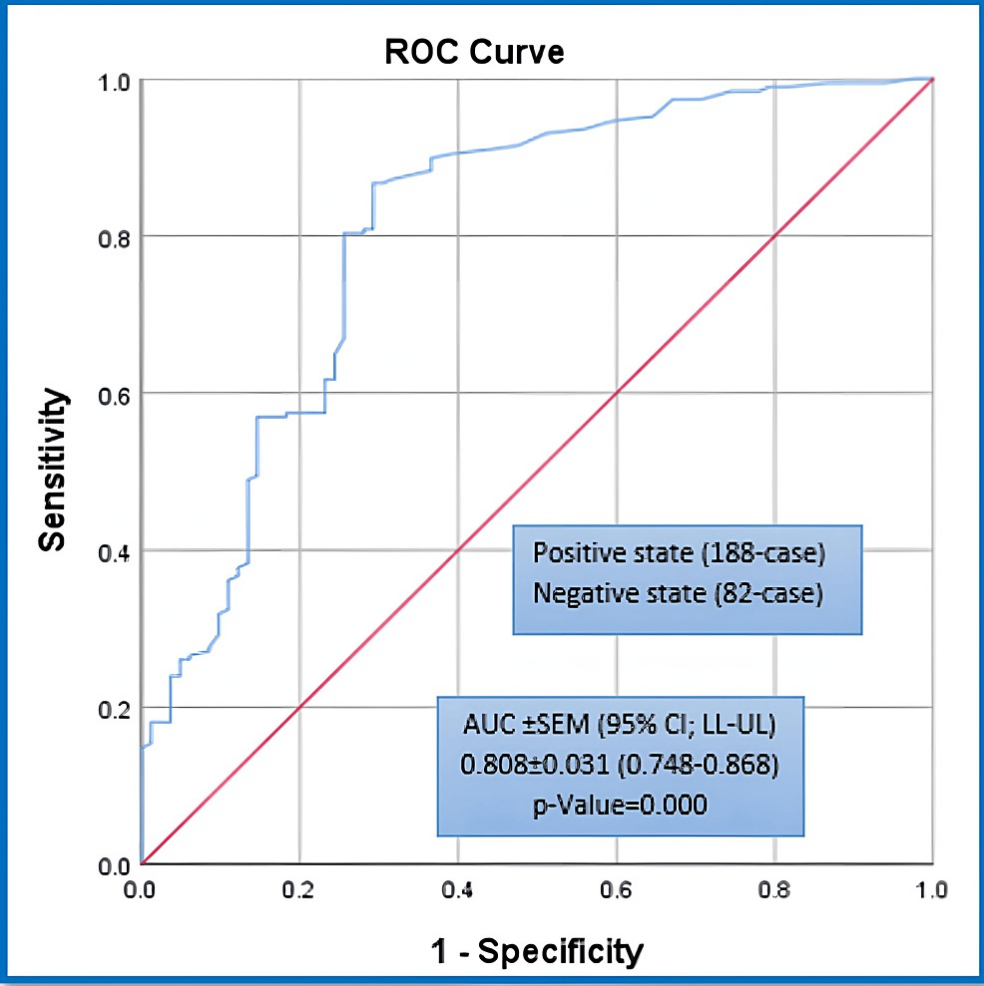
ROC analysis for NALR against the probability of adverse OI. ROC: receiver operating characteristic; NALR: neutrophils to albumin and lymphocytes ratio; OI: outcome of interest; AUC: area under the curve; SEM: standard error of the mean; LL: lower limit; UL: upper limit.

**Figure 2:**
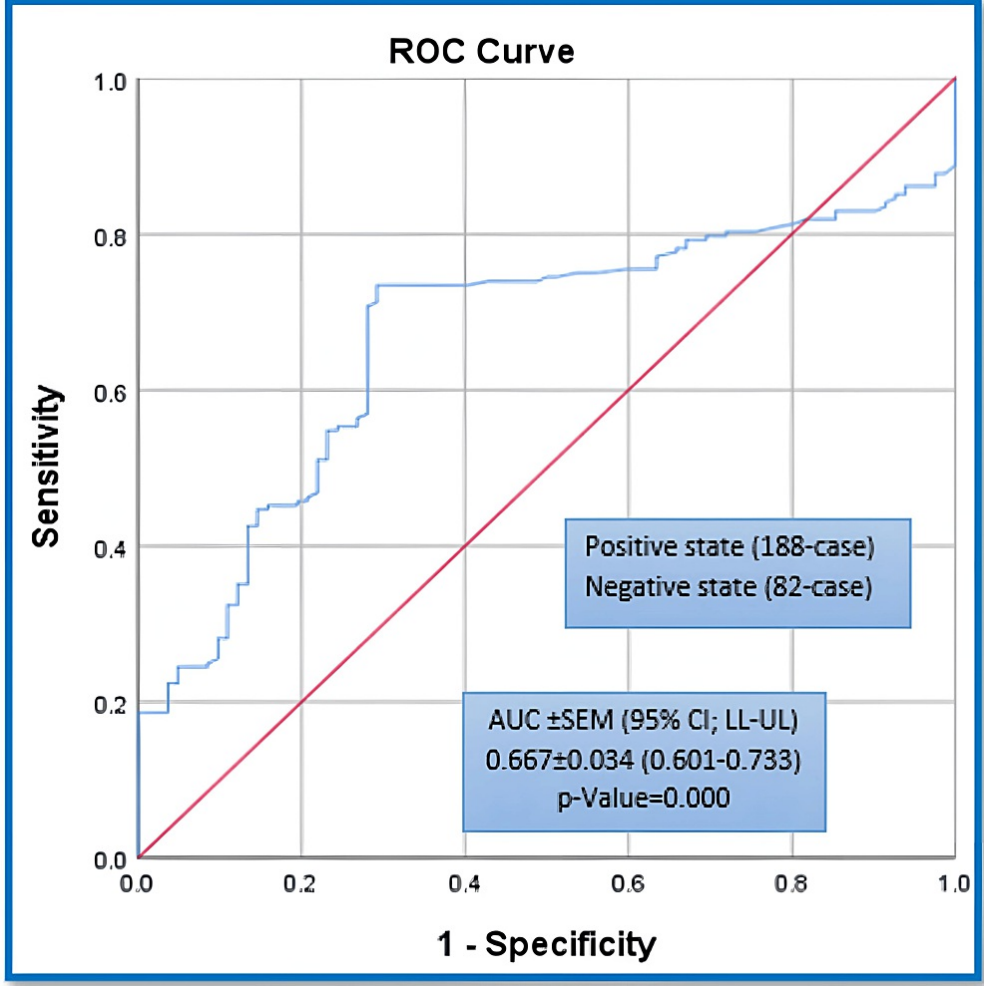
ROC analysis for NLR against the probability of adverse OI. ROC: receiver operating characteristic; NLR: neutrophils-to-lymphocytes ratio; OI: outcome of interest; AUC: area under the curve; SEM: standard error of the mean; LL: lower limit; UL: upper limit.

When the ROC curve analyses were conducted for both NALR and NLR, as major comparative prognosticators in this study, against the probability for OI incidence, we revealed that the area under the ROC curves (AUROC±SEM [95% CI: LL-UL]) were estimated at (0.808±0.031 [0.748-0.868], p-value=0.000) and (0.667±0.034 [0.601-0.733], p-value=0.000), respectively. The ROC curve test results are illustrated in Figures 1, 2.

Simultaneously, the binary logistic regression analyses stated that the estimated risks for NALR and NLR were 3.958 (95% CI: 2.564-6.109) and 1.202 (95% CI: 1.114-1.296), respectively. The abstracted coefficients that significantly explored the degree of correlations and predicted the probabilities for OI incidences were 1.376±0.221 and 0.184±0.039, respectively. The BLgR models were constructed as e(-2.047+1.376×NALR)/[1+ e(-2.047+1.376×NALR)] for NALR prognosticator and as e(-0.638+0.184×NLR)/[1+ e(-0.638+0.184×NLR)] for the NLR prognosticator. Indeed, the ranges of the total variations in the investigated probability of adverse OI and the % of cases that can be explained by the aforementioned constructed models were significantly determined at [19.1-27.1%, 81.5%, 39.929 (8), p-value<0.001] and [10-14.2%, 68.9%, 59.025 (8), p-value<0.001]. The BLgR analysis results are fully expressed in Table 3.

**Table 3:** BLgR analyses results for patients’ NALR and NLR against the probability of adverse OI. OI: outcomes of interest; B: abstracted coefficient; SEM: standard error of the mean; NLR: neutrophils-to-lymphocytes ratio; NALR: neutrophils to albumin and lymphocytes ratio; χ^2^: chi-square statistic; df: degree of freedom; VR: variation ranges; CI: confidence interval; EXP (B): exponential of the B and represents odds ratio or estimated risk. *p<0.05.

Tested Predictors	B±SEM	Wald	Significance	Exp(B)	95% CI for EXP(B)		χ2 (df), p-Value	VR	% Cases
					Lower	Upper			
Positivity OI									
Constant	-2.047±0.442	21.465	0.000	0.129			39.929 (8), 0.000*	19.1-27.1%	81.5%
NALR	1.376±0.221	38.603	0.000	3.958	2.564	6.109			
Positivity OI									
Constant	-0.638±0.313	4.154	0.042	0.529			59.025 (8), 0.000*OI	10-14.2%	68.9%
NLR	0.184±0.039	22.754	0.000	1.202	1.114	1.296			

The optimal operating thresholds for both tested poorer OI prognosticators, NALR and NLR, were explored at 1.5 and 5.37, respectively. The accompanied sensitivity indices that were adjunctively abstracted from the conducted sensitivity analyses were 86.7% versus 73.4%, 70.73% versus 70.73%, and 74.60% versus 71.38% for the sensitivities, specificities, and accuracy indices, respectively. The other sensitivity indices results are totally presented in Table 4.

**Table 4:** The optimal cutoff points and their corresponding sensitivity indices for our investigated prognosticators; NALR and NLR against adverse OI. OI: outcomes of interest; TPR: true positive rate (sensitivity); FPR: false positive rate; YI: Youden index; TNR: true negative ratio (specificity); PPV: positive predictive value; NPV: negative predictive value; AI: accuracy index; NLR: negative likelihood ratio; %OI, probability of adverse OI at explored optimal cutoff point.

Variables	Cutoff	TPR	FPR	YI	TNR	PPV	NPV	NLR	AI	OI
NALR	1.5	86.7%	29.3%	57.43%	70.73%	48.61%	94.34%	18.80%	74.60%	50.42%
NLR	5.37	73.4%	29.3%	44.14%	70.73%	44.47%	89.28%	37.60%	71.38%	58.66%

**Figure 3:**
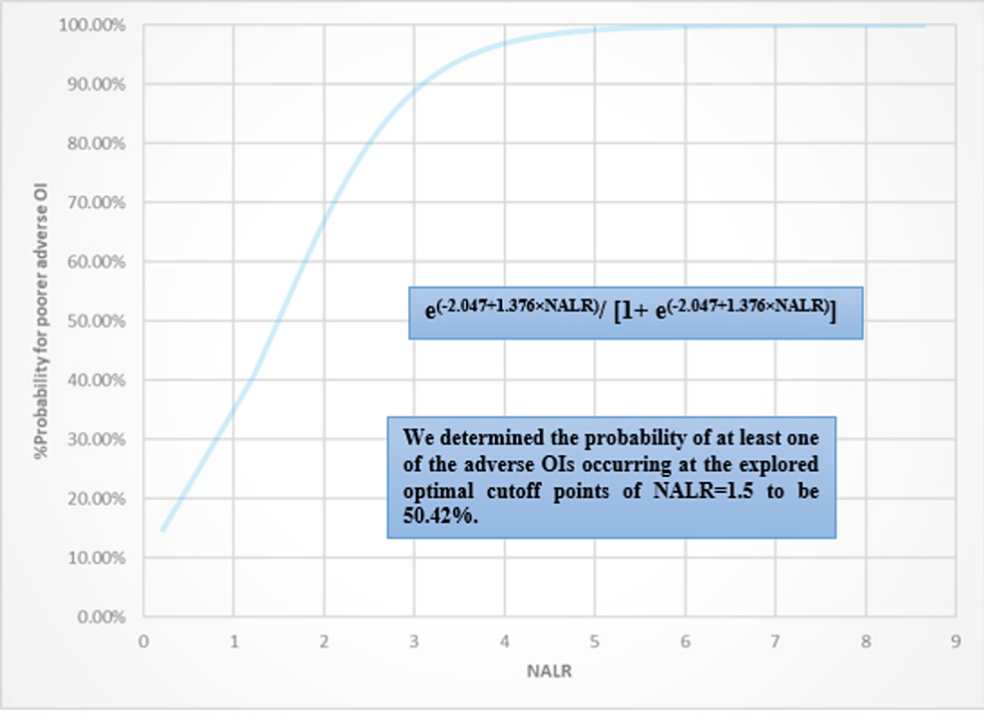
BLgR illustration for NALR against the probability of adverse OI. BLgR: binary logistic regression analysis; NALR: neutrophils to albumin and lymphocytes ratio; OI: outcome of interest.

**Figure 4:**
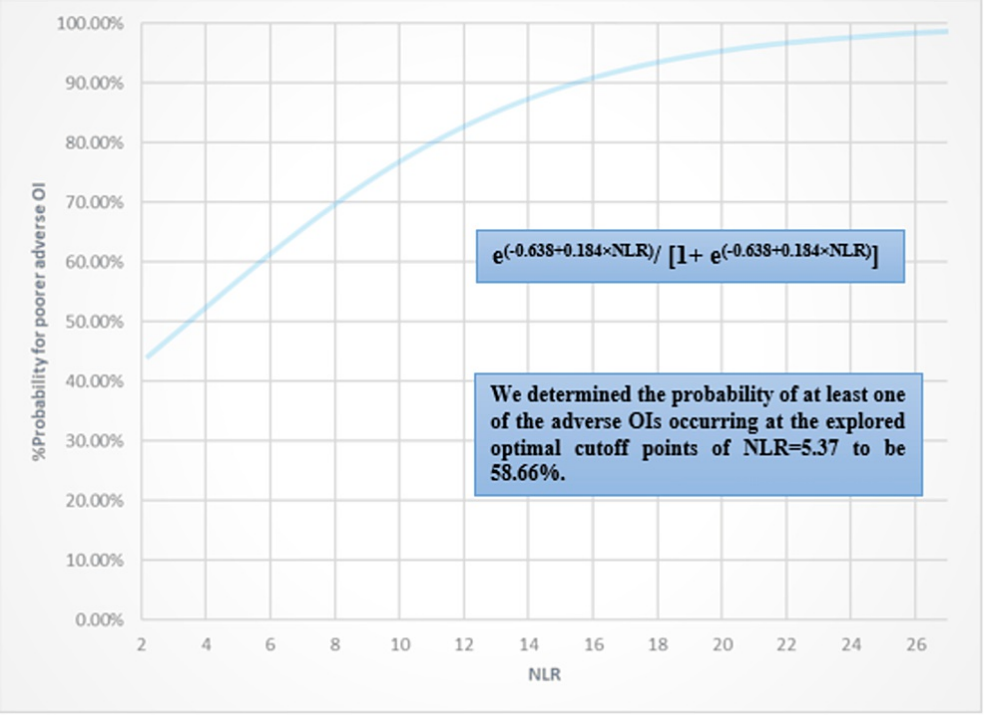
BLgR illustration for NLR against the probability of adverse OI. BLgR: binary logistic regression analysis; NLR: neutrophils-to-lymphocytes ratio; OI: outcome of interest.

Additionally, the probability of at least one of the adverse OIs occurring at the explored optimal cutoff points of NALR at 1.5 and NLR at 5.37 was 50.42% and 58.66%, respectively. The BLgR correlations for the NALR and NLR against the adverse OI are illustrated in Figures 3, 4.

While the surgical cohort had a higher number of participants compared to the medical cohort, the distribution rates between the two cohorts, specifically the better OI cohort (Group I) and the poorer OI cohort (Group II), were not statistically significant (p-value=0.527). Furthermore, due to the corticosteroidal effects, there is an increase in neutrophil levels and a decrease in lymphocyte levels, resulting in an exaggerated NLR and potentially the NALR, leading to overestimated interpretations. To assess the clinical usefulness of our main tested NALR prognosticator, we utilised the Acute Physiological and Chronic Health Evaluation II (APACHE II) and examined its ability to predict unfavourable OIs through a multiple regression analysis. Therefore, we performed a multiple logistic regression analysis to examine the relationship between our main prognostic factor of interest, NALR, and the potential confounding factors of corticosteroid use (specifically hydrocortisone equivalent), the type of ward (surgical versus medical), and the relevant prognostic factor APACHE II, in relation to the likelihood of poorer outcomes. Our analysis showed that the three integrated potential confounders had statistically insignificant effects, except for NALR which had a statistically significant effect with a coefficient value standard error of 0.034±0.002. This translates to an estimated risk of 1.034 (95% CI: 1.029-1.039) (Table 5).

**Table 5:** A multiple logistic regression analysis results. NALR: neutrophils to albumin and lymphocytes ratio; HC eq: hydrocortisone equivalent; APACHE II: Acute Physiological and Chronic Health Evaluation II.

Tested Predictors	B±SEM	Wald	df	Significance	Exp(B)	95% CI for EXP(B)	
						Lower	Upper
NALR	0.034±0.002	184.915	1	0.000	1.034	1.029	1.039
Ward (medical vs surgical)	-0.229±0.217	1.109	1	0.292	0.795	0.519	1.218
HC eq (mg/day)	0.011±0.009	1.354	1	0.245	1.011	0.993	1.029
APACHE II	-0.009±0.025	0.114	1	0.735	0.991	0.944	1.042
Constant	-7.929±1.087	53.243	1	0.000	0.000		

## Discussion

Leukocytes, dendritic cells, natural killer cells, and plasma proteins comprise the innate immune system. These components are the main pathogen-fighters. Innate immunity reacts to any antigen, while adaptive immunity targets specific ones. Hospitalisation-induced systemic inflammatory responses greatly impact lymphocyte count, a marker of adaptive immunity [13].

Ageing lowers serum albumin concentration. In particular, older people have lower albumin levels. Men aged 71-74 have an average albumin level of 41.6 g/l. In men 90 years or older, it drops to 38.5 g/l. Women of the same age group have lower albumin levels, dropping from 41.1 to 38.9 g/l. Malnutrition, common in hospitalised patients, affects albumin and lymphocyte levels [14].

Hospitalised patients with various degrees of infection may experience hyperinflammatory responses due to immunoinflammatory processes involving immune cells and mediators. This is characterised by a significant difference between neutrophil levels and the product of lymphocyte count and albumin level, rather than solely using lymphocyte count [15].

Many risk estimation models have been developed for hospitalised patients to assess clinical significance. Neutrophils, lymphocytes, and albumin are considered in these models. They assess subclinical inflammation and predict medical and surgical outcomes [16].

In hospitalised cachexia patients, lymphocytes and albumin levels are linked. More severe critically ill patients may have had lower serum albumin levels during risk stratification than less severe patients or non-ICU patients. Hypoalbuminemia-related ICU admissions have been thoroughly explained. Chronic inflammation inhibits albumin synthesis, oncotic pressures decrease, and microvascular permeability increases due to chronic hypoxia [17].

Sejópoles et al. examined how hematologic biomarkers predict COVID-19 mortality in Cuiabá, Brazil. COVID-19 exemplifies hyperinflammatory response syndrome and oxidative stress. The study by Sejópoles et al. included 199 patients. The study examined clinical and laboratory factors linked to cardiovascular involvement and hospital death. Potential mortality indicators included neutrophils, lymphocytes, monocytes, NLR, and MRL. A correlation was found between mortality and leukocyte, neutrophil, and lymphocyte counts, as well as the NLR and MRL. Leukocyte, neutrophil, lymphocyte, NLR, and MLR counts were accurate. The study suggests these biomarkers could predict COVID-19 death in hospitalised patients [18].

Yu YY et al. examined the preoperative neutrophil-to-albumin ratio (NAR) in 622 oral squamous cell carcinoma patients for prognostic value. To determine survival factors, the Cox proportional hazards model was used. The best NAR threshold for overall survival prediction was 0.1. High NAR independently predicted poor overall survival. Preoperative NAR is a convenient and effective oral squamous cell carcinoma prognostic indicator, according to the study [19].

Jiao JB et al. examined the diagnostic efficacy of serum C-reactive protein (CRP), erythrocyte sedimentation rate (ESR), globulin (GLB), albumin-to-globulin ratio (A/G), and NLR in PJI. One hundred and fifteen people with chronic PJI or aseptic loosening were studied. We collected data from January 2017 to December 2020. The study compared preoperative GLB, ESR, CRP, NLR, and A/G values for PJI diagnosis sensitivity and specificity. The median NLR levels in the PJI and aseptic groups were 2.510 and 1.850, respectively. CRP, ESR, and NLR had lower AUC values than CRP or ESR: 0.841, 0.850, and 0.708. Jiao JB et al. identified PJI with a diagnostic threshold of 2.1 or higher. The NLR had similar sensitivity (73.58%) and specificity (70.97%) to CRP and ESR, according to the author [20].

In a separate study, Lan CC et al. examined the prognostic value of hematologic inflammatory biomarkers, specifically the neutrophil-percentage-to-albumin ratio (NPAR), NLR, and ELR, in predicting COPD mortality. After adjustment, higher NLR was associated with higher all-cause and cardiovascular disease (CVD) mortality in 1158 subjects. Albumin reduced CVD and all-cause mortality. No correlation existed between lymphocyte levels and mortality. The nonparametric autoregressive (NPAR) and NLR recalibrated AUC values for five-year all-cause mortality prediction were 0.808 and 0.799, respectively [21].

The neutrophils to lymphocytes ratio to albumin ratio (NLRAR) and white blood cell to haemoglobin ratio (WHR) were examined for prognostic significance in curative resected hepatocellular carcinoma (HCC) patients by Shen XA et al. Our study resembles this. Shen XA et al. adopted NLRAR, a medical abbreviation similar to NALR. However, the researchers found that the high NLRAR group had a lower overall survival rate. The NLRAR and WHR independently predicted survival. In stage I HCC patients, NLRAR and WHR may be prognostic indicators [22].

Unfortunately, we could not find a study that compared NALR and NLR in hospitalised patients without a cancer history. Our study is unique in that it compares the derived proposed NALR prognosticator to the parental NLR prognosticator in predicting adverse outcomes like length of stay and mortality. Our study examined the NALR prognosticator as a new risk stratification criterion. We examined the NALR and NLR's predictive abilities. OI dichotomisation was used to compare these abilities between groups. The study estimated that the area under the ROC curves for NALR was higher than in NLR. The optimal operating thresholds for NALR and NLR prognosticators were 1.5 and 5.37, respectively, with sensitivities, specificities, and accuracy indices of 86.7%, 70.73%, and 74.60%, respectively.

This study has many flaws. The retrospective study prevented causality between NALR and NLR and the poor OIs. The present study focused on hospitalised patients, limiting its relevance to outpatients. Outpatients have less severe symptoms than inpatients, especially those with recent infections. Corticosteroids and other hospital events can affect NALR and NLR levels. Prospective multicentre randomised trials should determine whether NALR is better than the parent NLR for diagnosing, triaging, and prognosticating medically and surgically admitted patients and adjusting hospitalisation strategies.

## Conclusions

The results of our study showed that the predictive performance of NALR was found to be better than its parent NLR prognosticator. This was indicated by a higher predictive area of 0.808±0.031 compared to 0.667±0.034 in predicting the poorer adverse OIs. NALR also demonstrated higher sensitivity, negative predictive value, and accuracy index. The prognosticators for both tested outcomes exhibited significantly higher rates and levels in the poorer OIs compared to the better OIs. Both NALR and NLR predictive tools can be used in clinical practice with optimal thresholds of 1.5 and 5.37, respectively. These tools help prioritise the clinical and pharmacotherapeutic care of hospitalised patients. The thresholds are based on the likelihood of patients experiencing unfavourable outcomes, which are proposed to be higher than 50.42% and 58.66% when the corresponding thresholds are exceeded.
